# Thermodynamic model for methanesulphonic acid recovery by tri-*n*-butyl phosphate

**DOI:** 10.1039/d6ra00938g

**Published:** 2026-04-07

**Authors:** Rayco Lommelen, Charlotte Lempereur, Koen Binnemans

**Affiliations:** a KU Leuven, Department of Chemistry Celestijnenlaan 200F, P.O. Box 2404 B-3001 Leuven Belgium rayco.lommelen@kuleuven.be

## Abstract

A unified, semi-empirical thermodynamic model for liquid–liquid equilibria was developed within the OLI Mixed-Solvent Electrolyte (MSE) framework to describe the solvent extraction (SX) of methanesulphonic acid (MSA) by tri-*n*-butyl phosphate (TBP) in aliphatic diluents, based on experimental data obtained for this study. The model accounts for non-ideality in both phases and captures the key TBP protonation and molecular MSA extraction mechanisms. The model reproduces MSA Gibbs free energy of transfers, extraction efficiencies, organic-phase density, volume change, and water uptake across 0.1–9.9 mol L^−1^ MSA, 50–100 wt% TBP, and 24–77 °C, and captures exothermic behaviour (standard state enthalpy of transfer is −20.6 ± 0.9 kJ mol^−1^). Validation with data on MSA extraction from aqueous nickel(ii) methanesulphonate solutions demonstrates robustness under realistic conditions. Process simulations of counter-current mixer-settlers predict that an overall MSA recovery of 94% could be achieved from a 1.5 mol L^−1^ solution of Ni(CH_3_SO_3_)_2_ with 1 mol L^−1^ excess MSA at realistic process conditions, enabling the recovery and recycling of MSA. This validated model provides a practical, predictive basis for designing and optimising MSA recovery in industrial hydrometallurgical flowsheets. Comparison of the experimental MSA extraction data with literature data on other strong acids reveals the following order of extraction efficiency by TBP: HNO_3_ > HCl ≈ MSA > H_2_SO_4_.

## Abstract 2

Protonation and molecular transfer are modelled from new experimental data, enabling predictive simulation of up to 94% acid recycling from a 1.5 mol L^−1^ nickel methanesulphonate solution.

## Graphical abstract

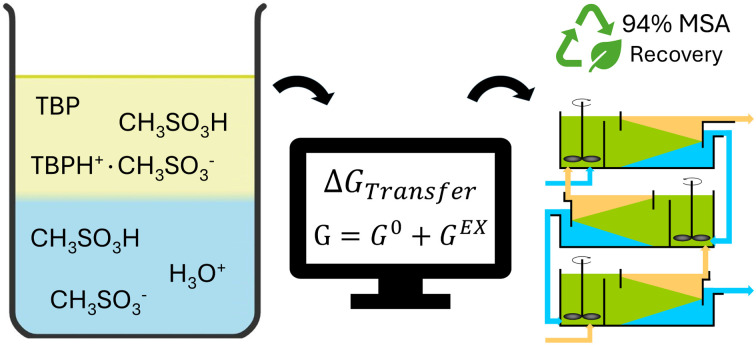

## 1 Introduction

Methanesulphonic acid (CH_3_SO_3_H, MSA) is a promising alternative to sulphuric acid for the development of circular hydrometallurgical processes.^1^ Among others, MSA shows great potential in lead, copper, zinc, and silver hydrometallurgy and in leaching of lithium-ion batteries.^1,2^ MSA is the smallest sulphonic acid molecule and is a very strong organic acid (p*K*_a_ = −1.9) with high solubilities of all its salts.^3^ Furthermore, it combines high electrochemical stability with sustainable properties like biodegradability, low volatility, and low toxicity.^4^ Crucial to the development of circular processes is the recovery or regeneration of the acid used in steps like leaching, electrowinning, and steel pickling. Typical routes include direct regeneration of MSA during electrowinning,^3,5^ regeneration through electrodialysis, as studied for Na_2_SO_4_,^6–8^ or recovery through solvent extraction (SX).^9–11^ Regeneration through pyrohydrolysis is not applicable to MSA due to its high boiling point (with decomposition) and negligible vapour pressure.^12^

SX is an important unit operation for acid recovery and metal separations because it couples high selectivity with scalable, counter-current operation. The method partitions an acid between an aqueous and an organic phase by interaction of the acid with extractant molecules in the organic phase.^13^ Nevertheless, these systems are chemically complex. Hydration, ion pairing, complexation, aggregation, and even third-phase formation can influence equilibria, loading, and phase behaviour.

Two SX systems have been studied by previous authors for the recovery of MSA: trioctylamine (TOA) and tris(2-ethylhexyl)amine (TEHA), with or without 1-octanol as phase modifier.^14,15^ Both show strong extraction of MSA, but this hinders effective stripping. Tri-*n*-butyl phosphate (TBP) is also known to extract acids, and its lower basicity improves the stripping efficiency.^9,16^ Additionally, TBP is widely used in the industry and is relatively inexpensive compared to TOA and TEHA. Similar to the extraction with TEHA and TOA, TBP can be protonated to form TBPH^+^ in strongly acidic media, enabling ion-pair extraction of the conjugate base (eqn (1)).^16^ When TBP is fully protonated, acid can also be extracted in its molecular form (eqn (2)). In both equations, subscript a represents aqueous phase and o stands for organic phase. As a result of this extraction mechanism, the stoichiometric ratio of MSA to TBP in the organic phase can exceed one.

1 CH_3_SO_3_H_a_ + TBP_o_ ⇋ ( TBPH^+^)(CH_3_SO_3_^−^)_o_

2 CH_3_SO_3_H_a_ ⇋ CH_3_SO_3_H_o_

Despite the relevance of SX of MSA, predicting SX performance across a wide range of conditions remains challenging.^17^ Furthermore, there are no experimental data, except for limited volume change and water uptake measurements.^18^ There exists no predictive thermodynamic model for MSA recovery by SX with TBP. This limits the ability to design efficient, closed-loop flowsheets for MSA recovery without extensive trial-and-error experimentation. Models based solely on equilibrium constants are insufficient for predicting equilibria in electrolyte systems with substantial non-ideality and complex speciation.^19^ Conversely, first-principles descriptions are computationally too demanding for calculating an accurate SX efficiency in mixed-solvent solutions containing high concentrations of electrolytes.^20–22^ Therefore, a semi-empirical thermodynamic approach offers a pragmatic compromise: experimental data define and constrain interaction parameters embedded within a rigorous activity-coefficient framework.

To construct a semi-empirical thermodynamic SX model, Pitzer-type equations for the aqueous phase are often combined with an organic phase that is either assumed ideal or activity-corrected by separate equations.^23–26^ This neglect of organic phase non-ideality or the absence of a unified thermodynamic framework limits the applicability of these models to real-world applications with high extractant loadings. Alternative approaches, such as mean-spherical approximation-NRTL hybrids or PC-SAFT-based equations of state, are promising.^27–30^ However, these lack integrated, user-friendly platforms and do not incorporate speciation and density corrections required for simulating SX processes.

The Mixed-Solvent Electrolyte (MSE) framework implemented in the OLI Systems suite provides a unified, semi-empirical approach that enables the calculation of SX systems with high extractant loadings.^31,32^ It computes phase and chemical equilibria by minimising the Gibbs free energy (Δ*G*) of the system. This Δ*G* combines standard state thermodynamic values of each species (*i*), resulting in a standard state Gibbs free energy of formation (Δ*G*^0^_f_(*i*)), with activity coefficients derived from an excess Gibbs free energy expression (Δ*G*^EX^). Δ*G*^EX^ can be decomposed into a sum of three contributions: (1) long-range electrostatic interactions described by the Pitzer–Debye–Hückel equation, (2) short-range molecular interactions captured by UNIQUAC, and (3) mid-range ion–ion and ion–molecule interactions represented by a second-virial-type expression. Interaction parameters for the latter two terms are fitted to experimental data. The MSE framework also incorporates density and excess volume correlations, which are essential for converting mole-fraction-based calculations into practical units such as mol L^−1^ or g L^−1^.

In this paper, we present experimental SX data for the MSA–H_2_O–TBP–dodecane system and use these results to develop a thermodynamic model based on the OLI-MSE framework. This model is subsequently validated against more realistic systems containing Ni(CH_3_SO_3_)_2_. Building on the acid–TBP extraction model developed by Lommelen *et al.*,^16,33^ the present work introduces additional interaction parameters specific to MSA chemistry. Further details on the application of the OLI-MSE approach to SX equilibria are provided in these earlier publications.

This work focuses on the development of an equilibrium thermodynamic model. Although viscosity and hydrodynamics can influence mass-transfer rates in practical equipment, these effects are not considered here. The thermodynamic model nevertheless provides the fundamental equilibrium information required for process design and for future rate-based or dynamic modelling that strongly depend on equipment and operation-specific choices in specific applications.

## 2 Experimental

### 2.1 Chemicals

Methanesulphonic acid (MSA, ≥99.5%) was obtained from Carl Roth GmbH (Karlsruhe, Germany). Tri-*n*-butyl phosphate (TBP, 99%), isopropanol (99%), potassium hydroxide (0.1 mol L^−1^), potassium hydroxide (0.01 mol L^−1^), pH buffers with pH values of 1, 4, 7 and 12, and 1000 ppm standard solutions of nickel and scandium in a 2–5% nitric acid matrix were obtained from Chem-Lab NV (Zedelgem, Belgium). Nitric acid (70%) and *n*-dodecane (99%) were obtained from Acros Organics (Geel, Belgium). Nickel(ii) carbonate, basic hydrate (99.9%) was obtained from Sigma-Aldrich GmbH (Steinheim, Germany). Sodium oxalate was obtained from VWR BDH Chemicals (Fontenay-sous-Bois, France). Imidazole was purchased from Fischer Scientific (Loughborough, United Kingdom). HYDRANAL™ Methanol and Composite 5 Honeywell were obtained from VWR International GmbH (Darmstadt, Germany). Sodium tartrate dihydrate and potassium hydrogen phthalate were purchased from Merck Millipore (Darmstadt, Germany). All water used was ultrapure water obtained with a Merck Millipore Milli-Q™ Reference Ultrapure Water Purification System. All chemicals were used as received, without any further purification.

### 2.2 Thermodynamic software

The thermodynamic model and all related calculations were developed using OLI Systems software (Parsippany, NJ), version 12.0. OLI Databook was employed to navigate the private OLI databases and to add or modify interaction parameters within the MSA–TBP database. Model files required for parameter optimisation were generated using OLI Chemistry Wizard, while OLI Regression Console was used to perform iterative regressions and fit interaction parameters to experimental data. After optimisation, the general OLI-MSE database and the finalised private databases were loaded into OLI Studio and OLI Flowsheet for equilibrium calculations. OLI Studio was used for batch-type calculations, enabling detailed analysis of the SX equilibria, whereas a whole counter-current, continuous SX process in mixer-settlers was designed in OLI Flowsheet to evaluate MSA recovery performance under operation scenarios.

### 2.3 SX experiments

SX experiments were conducted by mixing 5 mL of aqueous feed with 5 mL of an organic phase in 20 mL glass vials for 30 min at 200 rpm using a Thermoshake THL 500/1 from Gerhardt. This results in an initial organic-to-aqueous volumetric phase ratio (O/A_in_) of 1. The temperature was set to 25 °C unless otherwise stated. After shaking, the glass vials were centrifuged, and the two phases were separated. Aqueous feed solutions were prepared by diluting a known mass of acid to the desired volume. For the organic phases containing diluted TBP, a known mass of TBP was diluted in *n*-dodecane to a fixed volume.

For the validation series, a nickel(ii) methanesulphonate (Ni(CH_3_SO_3_)_2_) stock solution with a concentration of 2.1 mol L^−1^ was prepared by weighing nickel carbonate basic hydrate (NiCO_3_·2Ni(OH)_2_·*x*H_2_O, 28.71 g) in a round-bottom flask, and slowly adding MSA (30.15 mL) and water (69.85 mL). The 10% excess of nickel(ii) carbonate basic hydrate ensured that all MSA had reacted after stirring overnight at 200 rpm. Then, the leftover nickel carbonate basic hydrate was filtered on a Büchner filter, and the nickel content was measured in quintuplicates using Inductively Coupled Plasma Optical Emission Spectroscopy (ICP-OES) on a PerkinElmer Optima 8300 instrument. Finally, SX experiments were performed by adding a certain amount of MSA to the Ni(CH_3_SO_3_)_2_ stock solution and mixing it with an organic phase. This SX procedure was performed in the same manner as described above, with the exception that graduated 15 mL centrifuge tubes were used instead of 20 mL glass vials. This modification allowed for a direct determination of volume changes at equilibrium.

Acid–base titration experiments were performed to determine initial and equilibrium MSA concentrations on a T5 Automatic titrator from METTLER TOLEDO with an aqueous KOH solution (0.1 mol L^−1^). A DGi111-SC pH electrode (METTLER TOLEDO) was used for titrations on the aqueous phase, while a DGi113-SC pH electrode (METTLER TOLEDO) was employed for measurements in the organic phase. For titrations involving the organic phase, isopropanol was selected as the solvent, as preliminary solubility tests confirmed its compatibility with both the organic phase and the titrant. The water content in the organic phase at equilibrium was quantified *via* Karl Fischer titration using a volumetric V30S titrator (METTLER TOLEDO), with the sample introduced directly into the titration vessel. To maintain an optimal pH during the analysis, imidazole was added to the reactor. The density of the organic phase (*ρ*_o_) was determined using a DMA 4500 M density meter (Anton Paar). Equilibrium volume changes, and the corresponding organic-to-aqueous volume ratio (O/A)_e_, were assessed either visually using a graduated cylinder or calculated based on the complete composition of the organic phase in combination with its measured *ρ*_o_.

The distribution ratio (*D*) of MSA was calculated by dividing its molarity in the organic phase ([MSA]_o_) by that in the aqueous phase ([MSA]_a_). This value can be converted to the Gibbs free energy of transfer (Δ*G*_t_) to obtain a metric more closely related to thermodynamics:3
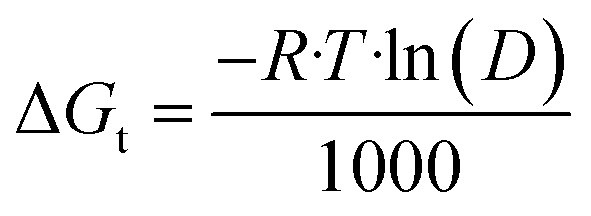
Δ*G*_t_ is expressed in kJ mol^−1^, with *R* being the universal gas constant (8.314 J mol^−1^ K^−1^) and *T* the temperature in Kelvin. The extraction efficiency (%*E*) of an acid was calculated as follows:4
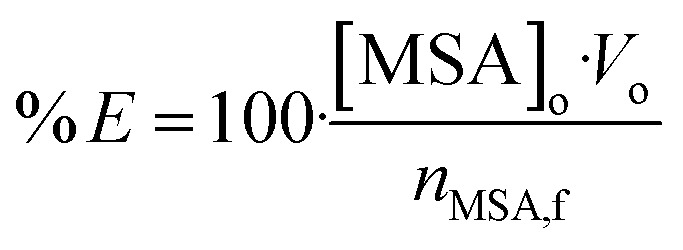
with *V*_o_ being the volume of the organic phase at equilibrium and *n*_MSA,f_ being the number of moles of MSA in the feed. The percentage loading (%*L*) of the extractant was expressed as:5
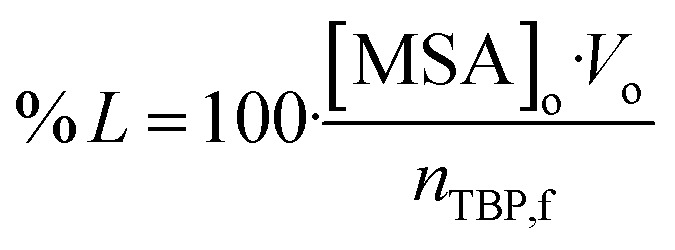


based on the hypothesis that the acid:TBP stoichiometry in the extracted species is 1 (*vide infra*).

## 3 Results and discussion

### 3.1 Experimental data

#### 3.1.1 Effect of MSA concentration

Only one experimental study on the MSA–TBP system could be found in the literature.^18^ The authors mention significant volume changes upon the extraction of MSA, due to the coextraction of four water molecules per MSA molecule. Comparable volume changes are observed in other strong acid–TBP extraction systems.^34^ The MSA–TBP dataset was significantly extended to understand the extraction mechanism and provide training and validation data for the thermodynamic model.

**Fig. 1:**
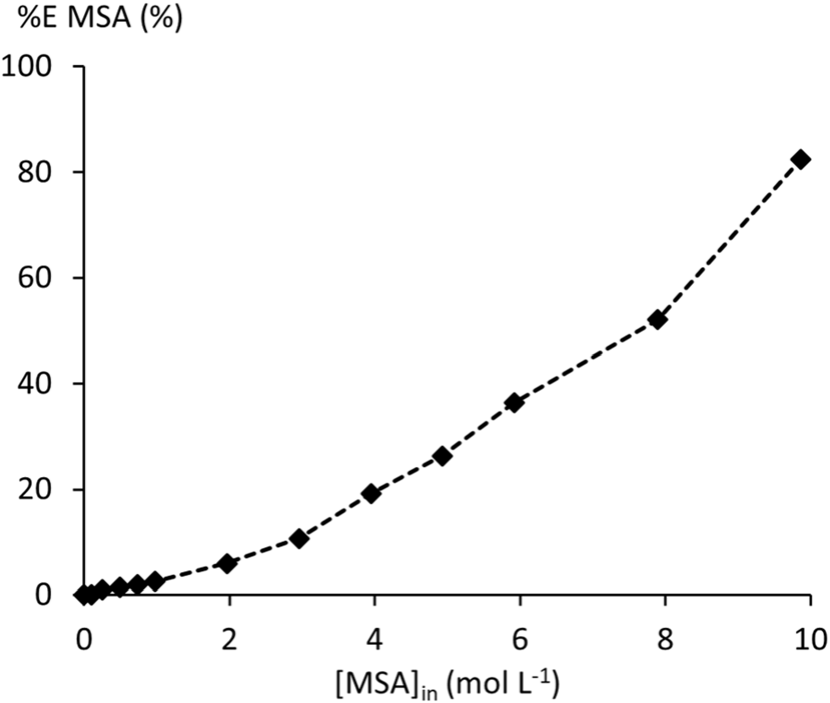
Extraction efficiency of MSA by 100 wt% TBP.

**Fig. 2:**
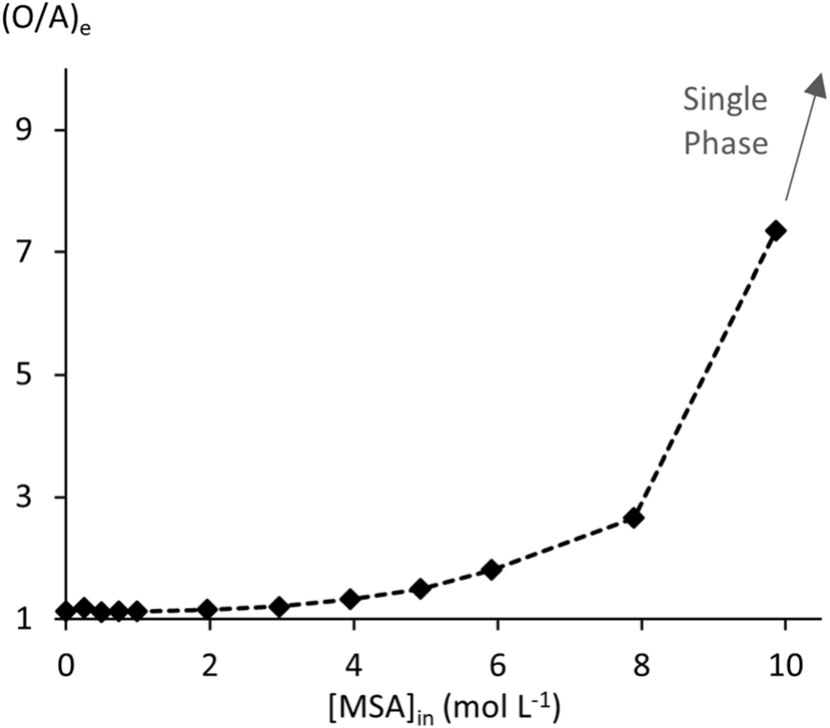
Equilibrium volume ratios for the MSA–100 wt% TBP system.

MSA extraction by undiluted (100 wt%) TBP was studied over an initial aqueous MSA concentration [MSA]_a,in_ the range from 0.10 to 15.04 mol L^−1^ at 25 °C. Full experimental details can be found in Table S1 of the SI. MSA extraction increases with [MSA]_a,in_ up to 83% at 9.86 mol L^−1^ [MSA]_a,in_ (Fig. 1). From 11.8 mol L^−1^ onwards, extraction of MSA and water resulted in a single phase (Fig. 2). The increase in (O/A)_e_ reflects both a reduction in aqueous phase volume and an increase in organic phase volume, leading to solute upconcentration in the raffinate.

**Fig. 3:**
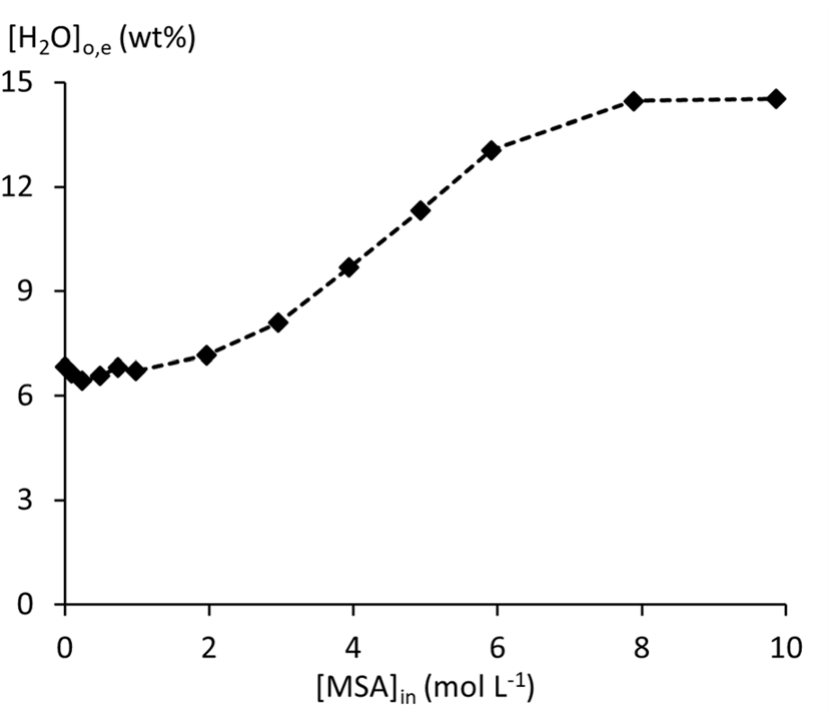
Equilibrium water uptake by 100 wt% TBP, contacted with MSA solutions.

The %*L* exceeds 100% at 7.89 mol L^−1^ [MSA]_a,in_, marking the point where the (TBPH^+^)(CH_3_SO_3_^−^) complex begins to extract molecular MSA (eqn (2)). [H_2_O]_o,e_ reaches a plateau at this point, while the acid concentration and %*L* further increase (Fig. 3). These observations indicate that the extraction of molecular MSA does not involve additional coextraction of water. The large and variable water content in the organic phase reveals strong non-ideal behaviour in the organic phase. In a thermodynamic model, this non-ideal behaviour cannot be ignored. Furthermore, the presence of a significant amount of water content in the organic phase indicates that a thermodynamic model with a single activity framework for both phases would provide the most accurate representation of the system.

#### 3.1.2 Effect of TBP concentration

Extractants are often diluted in a nonpolar solvent to lower the viscosity of the loaded organic phase for easier process operations, and to reduce *n* to avoid phase inversion and improve phase separation. Aliphatic diluents are generally preferred in SX processes over aromatic diluents because they are safer and more environmentally friendly, they have a smaller impact on extraction efficiency, a lower density, a lower toxicity, and produce less odour nuisance.^35,36^ Their main downside is a higher tendency to form third phases and emulsions, which can be reduced by adding modifiers such as long-chain alcohols. TBP itself is frequently used as a modifier in systems with other extractants.^37–39^

**Fig. 4:**
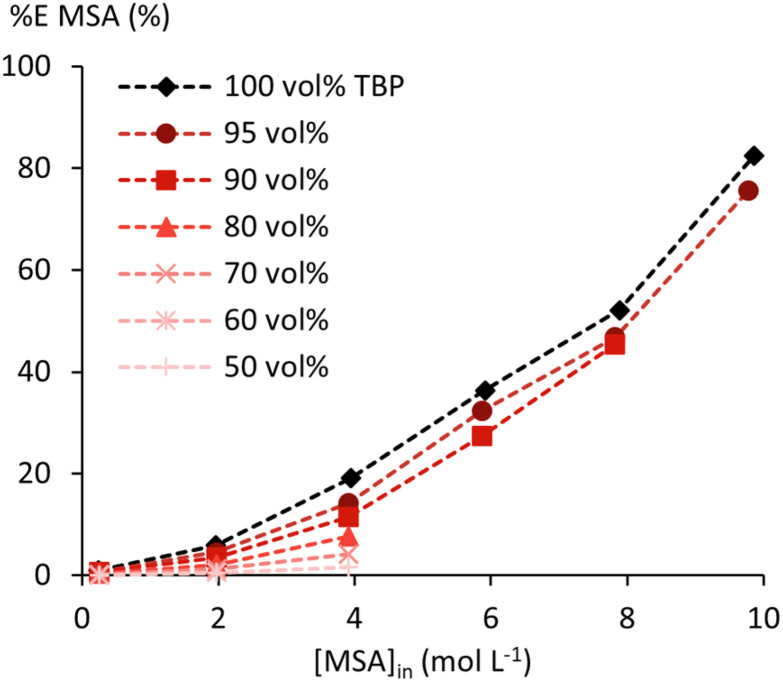
Extraction efficiency of MSA by 50 to 95 wt% TBP diluted in *n*-dodecane. Undiluted TBP data (black diamonds) from Fig. 1 are given for comparison.

In this study, *n*-dodecane was selected as the model diluent for the construction of the thermodynamic model. Any other aliphatic diluent used in processes or experiments can be substituted with *n*-dodecane for calculations with the presented thermodynamic model, as equilibrium properties such as extraction efficiency are largely independent of the specific aliphatic diluent.^40,41^ Practically, the aliphatic diluent used in the experiments or process can be replaced in the OLI calculation by *n*-dodecane without loss of accuracy. Table S5 in the SI can be used to convert a certain concentration of TBP in an aliphatic diluent from vol% or molarity to an amount of TBP and *n*-dodecane required to represent the solvent. To gather sufficient data to understand the effect of *n*-dodecane on the extraction of MSA by TBP and add these effects to the thermodynamic model, the extraction of MSA by 50 to 95 wt% TBP in *n-*dodecane was studied at 25 °C (Fig. 4 and SI Table S2).

**Fig. 5:**
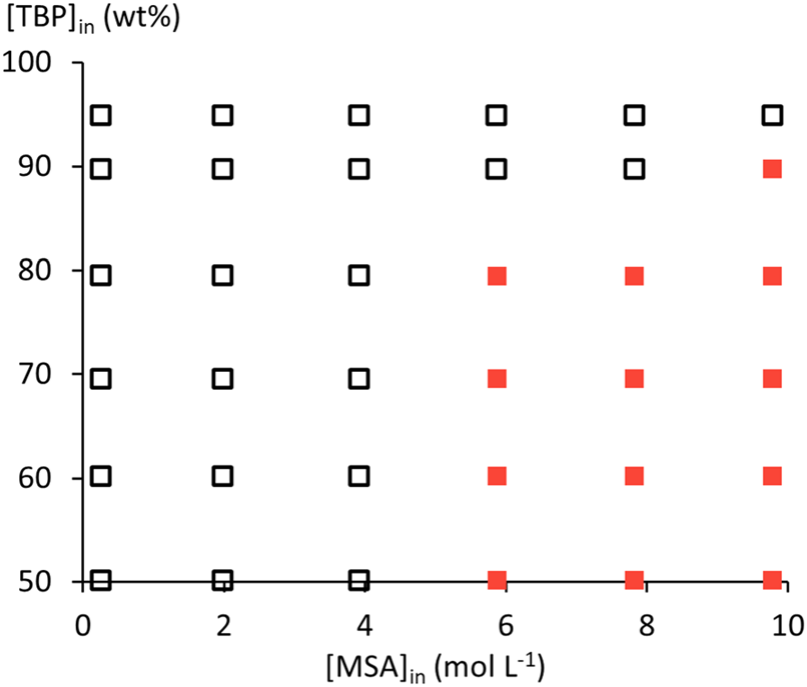
Single organic phase (□) or organic phase splitting (■) observed at equilibrium.

Extraction of MSA by TBP decreases more than stoichiometrically with increasing dilution in *n*-dodecane. This is also evident from the %*L* values in Table S2, which decrease with increasing TBP dilution for the same [MSA]_in_. Water uptake by the organic phase follows this same trend. Both indicate a destabilising effect of *n*-dodecane on the extracted (TBPH^+^)(CH_3_SO_3_^−^) adduct and the coextracted water, revealing repulsive non-ideal intermolecular interactions between *n*-dodecane and the other species in the organic phase. (O/A)_e_ increases with increasing MSA and TBP concentrations, but no phase miscibility is encountered until the highest studied [MSA]_in_ of 9.8 mol L^−1^. However, third-phase formation occurred at high MSA and low TBP concentrations, likely due to destabilisation of the (TBPH^+^)(CH_3_SO_3_^−^) adduct in the organic phase (Fig. 5).

#### 3.1.3 Effect of temperature

**Fig. 6:**
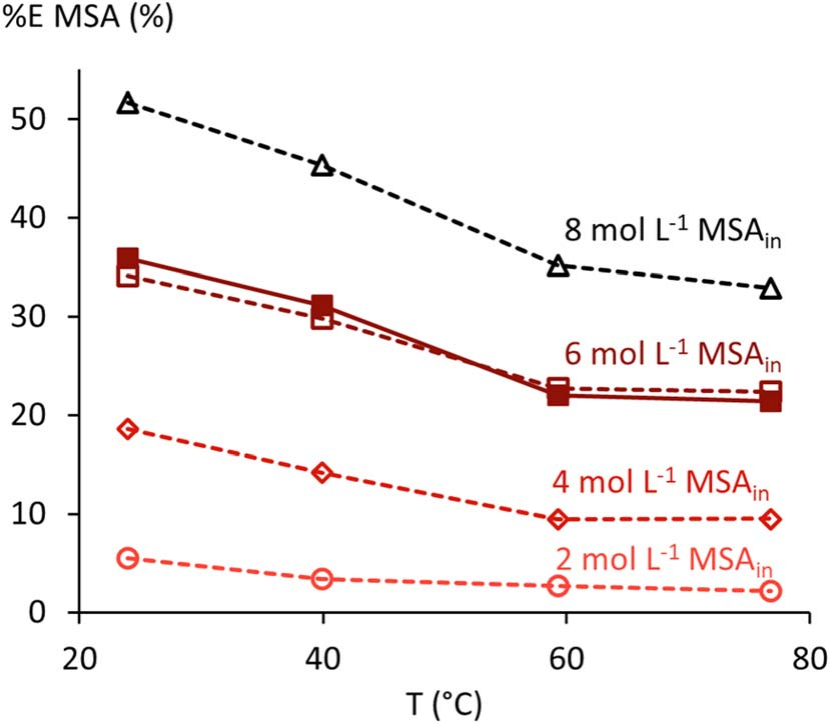
Extraction efficiency of MSA by undiluted TBP *versus* temperature (*T*). Filled markers show %*E* based on experimentally determined *V*_o_, while open markers show %*E* based on *V*_o_ values calculated by the thermodynamic model (see below).

Temperature is an important process control parameter for acid recovery by SX, as higher temperatures generally yield better acid stripping efficiency.^16,42^ This trend is also observed in SX experiments for MSA using undiluted TBP, where the influence of temperature (24–77 °C) and initial MSA concentration (2.0–8.0 mol L^−1^) was investigated (Fig. 6 and SI Table 3). Acid concentrations in both the aqueous and organic phase were determined by titration, enabling direct calculation of SX properties such as *D* and Δ*G*_t_, but volumetric data are required to calculate %*E* based on concentrations. Accurate volumetric data were available only for the series with 6 mol L^−1^ MSA in the feed, as this was the only series where the complete organic-phase composition (including water uptake) and *ρ*_o_ were measured. For the other series, *V*_o_ was calculated using the thermodynamic model (*vide infra*). In Fig. 6, a visual distinction is made between the %*E* values based on experimental (open markers) and calculated (filled markers) *V*_o_ data. A comparison between both approaches for the 6 mol L^−1^ series shows that only minimal deviations between the two approaches can be expected for the other series.

The Van ‘t Hoff equation (eqn (6)) enables estimating the extraction (transfer) enthalpy (Δ*H*_t_) and entropy (Δ*S*_t_) for the extraction of MSA by relating ln(*D*) to 1000/*T*, with *T* the temperature in Kelvin and *R* the universal gas constant (8.314 J mol^−1^ K^−1^). Its application assumes that Δ*H*_t_ and Δ*S*_t_ remain constant over the investigated temperature range, which is reasonable for the relatively narrow interval studied.6
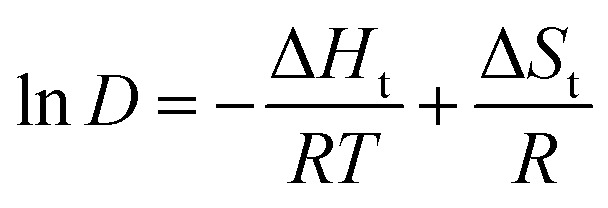


**Fig. 7:**
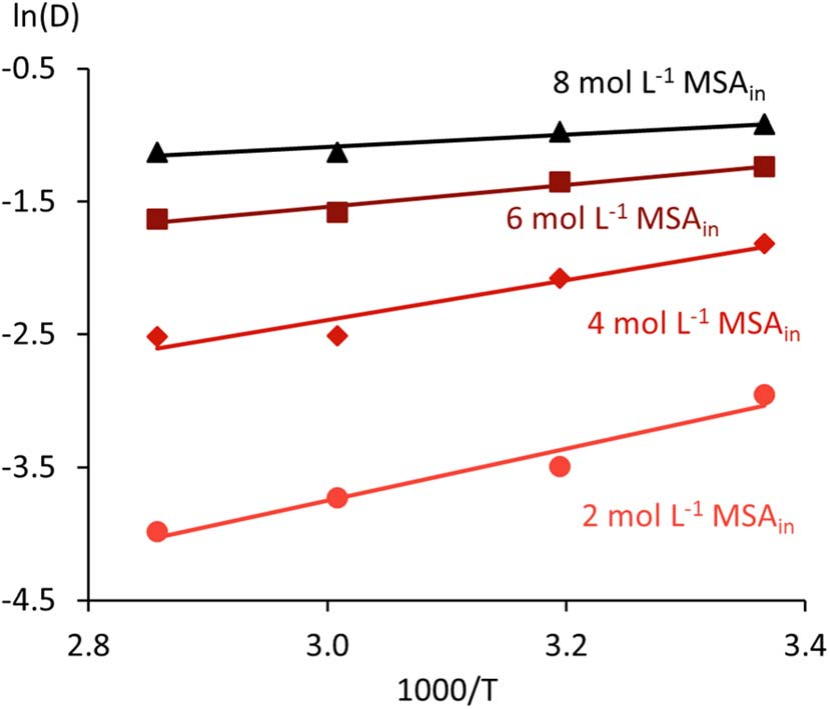
Van ‘t Hoff analysis of MSA extraction by undiluted TBP.

A graphical representation of this analysis can be found in Fig. 7, and its results are given in Table 1. The negative Δ*H*_t_ values for all initial MSA concentrations indicate the exothermic nature of the extraction of MSA. Both the exothermic contribution and the change in entropy decrease with increasing MSA concentration in the feed. For almost all conditions, the extractant loading remains below 100%, with %*L* exceeding 100% only for the 8 mol L^−1^ MSA_in_ series at 24 °C (114 %*L*) and 40 °C (101 %*L*). This indicates that a changing extraction mechanism, from protonation of TBP to extraction of molecular acid, can at most explain a small part of the change in thermodynamic values. The largest part of the differences should originate from effects due to the non-ideal behaviour of MSA in the aqueous phase and the (TBPH^+^)(CH_3_SO_3_^−^) adduct in the organic phase. A linear extrapolation of Δ*H*_t_ and Δ*S*_t_ towards infinite dilution results in a Δ*H*^0^_t_ of −20.6 ± 0.9 kJ mol^−1^ (*R*^2^ = 0.989) and a Δ*S*^0^_t_ of −95.7 ± 4.1 J mol^−1^ K^−1^ (*R*^2^ = 0.988).

**Table 1:** Results for the Van ‘t Hoff analysis of MSA extraction by undiluted TBP

[MSA]_in_, mol L^−1^	Δ*H*_t_, kJ mol^−1^	Δ*S*_t_, J mol^−1^ K^−1^	*R* ^2^
2.02	−16.2 ± 2.4	−77.2 ± 7.5	0.957
4.00	−12.5 ± 2.5	−57.3 ± 7.7	0.927
6.02	−6.9 ± 0.9	−33.5 ± 2.8	0.966
8.01	−3.8 ± 0.8	−20.6 ± 2.6	0.912

#### 3.1.4 Effect of nickel(ii) methanesulphonate

A typical hydrometallurgical stream that would contain a recoverable amount of MSA would also contain a significant concentration of dissolved methanesulphonate salts. To study the effects of such salts on the extraction of MSA by TBP, two series of experiments were performed with Ni(CH_3_SO_3_)_2_. Nickel(ii) is used here as a model ion for studying such effects, because (1) Ni(CH_3_SO_3_)_2_ is highly soluble in aqueous solutions,^43,44^ (2) the Ni(CH_3_SO_3_)_2_–MSA–water chemistry is available in OLI through a private database,^43^ (3) nickel is a commonly found in modern hydrometallurgical flowsheets, for instance for the production of nickel-containing cathode-active materials for Li-ion batteries.^45^

The extraction of 4.60 and 7.67 mol L^−1^ MSA by undiluted TBP was studied with a varying initial Ni(CH_3_SO_3_)_2_ concentration up to 1.47 and 1.10 mol L^−1^, respectively. These concentration limits were determined based on the solubility of Ni(CH_3_SO_3_)_2_ in 4.60 and 7.67 mol L^−1^ MSA, based on calculations performed in OLI Studio using a private Ni(CH_3_SO_3_)_2_ database.^43^ An overview of the initial and equilibrium properties for these two series can be found in the SI Table S4.

**Fig. 8:**
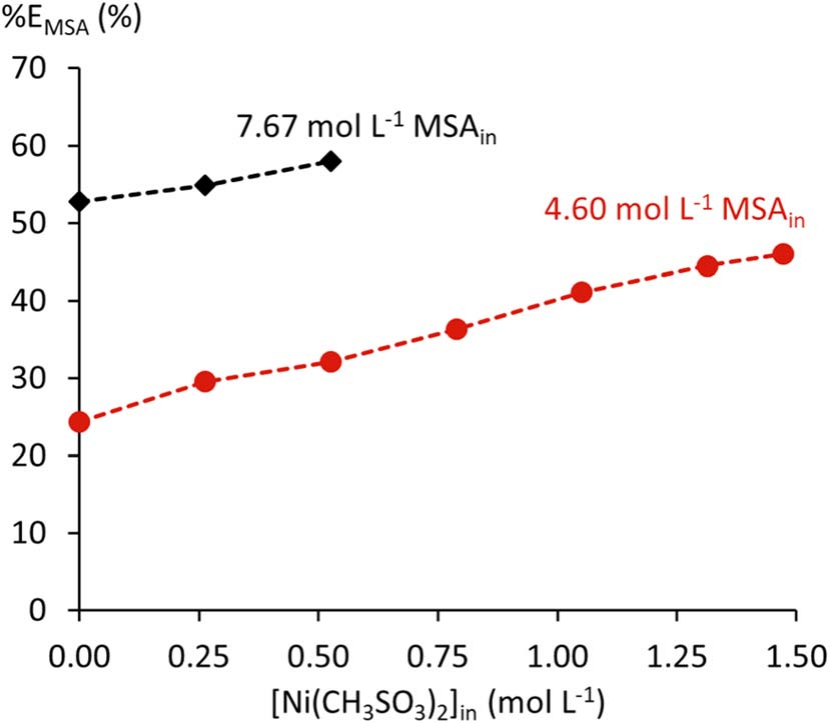
Extraction efficiency of MSA by undiluted TBP in the presence of Ni(CH_3_SO_3_)_2_.

The extraction of nickel(ii) by TBP was negligible for all tested conditions, based on the absence of the typical green colour for nickel(ii) in the organic phase. This is not surprising, as TBP typically extracts metal ions as neutral complexes through complexation with the anion. The nickel(ii) ion is strongly hydrated in the aqueous phase, and methanesulphonate is a weakly coordinating anion. The extraction of MSA increases slightly with increasing Ni(CH_3_SO_3_)_2_ content due to salting-out effects (Fig. 8). Nickel ions compete for water molecules, reducing MSA hydration and favouring its extraction. This is again a clear example of the non-ideal behaviour in the system.

An almost constant water content was found in the organic phase for the 4.60 mol L^−1^ MSA_in_ series, while the water content decreased with increasing Ni(CH_3_SO_3_)_2_ for the 7.67 mol L^−1^ MSA_in_ series (SI, Table S4). This seems to contradict the observations in Ni(CH_3_SO_3_)_2_-free systems, where the water content increases with increased MSA extraction. Likely, the hydration of the ions from Ni(CH_3_SO_3_)_2_ counteracts the coextraction of water.

A light green precipitate was formed from 0.79 mol L^−1^ Ni(CH_3_SO_3_)_2_ in the feeds with 7.67 mol L^−1^ MSA. Samples with 0.53 mol L^−1^ Ni(CH_3_SO_3_)_2_ or less did not show any precipitation. The appearance of this precipitate corresponds to that of hydrated nickel(ii) methanesulphonate.^43^ This precipitation likely results from a decrease in aqueous phase volume due to the coextraction of water to a point where the solubility limit of Ni(CH_3_SO_3_)_2_ is exceeded. The points where precipitation was observed were excluded from further analysis, due to a change in equilibrium aqueous Ni(CH_3_SO_3_)_2_ and water concentration because of the formation of hydrated Ni(CH_3_SO_3_)_2_ species.^43,44^

#### 3.1.5 Comparison with other acids

**Fig. 9:**
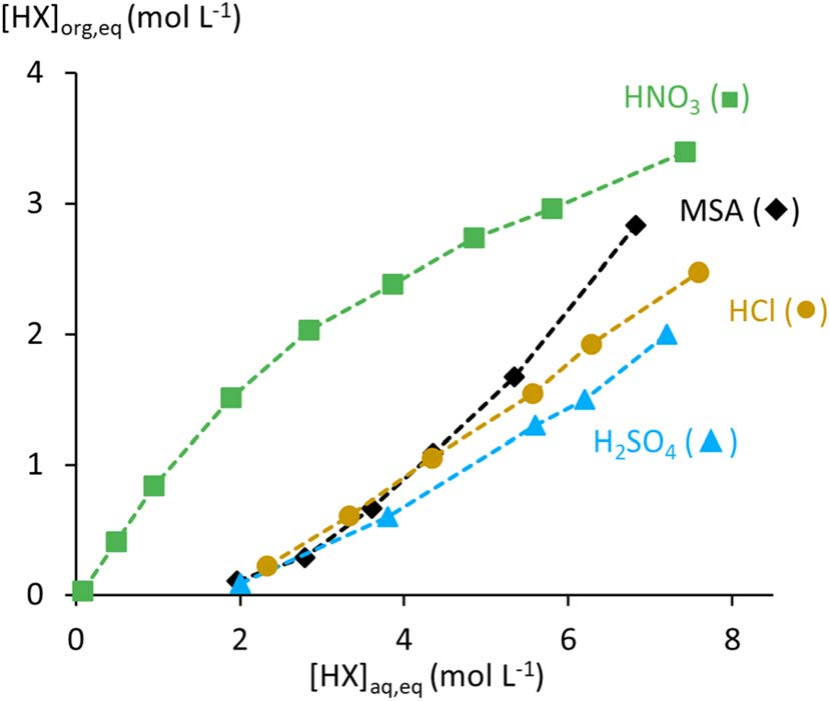
Distribution isotherms for the extraction of MSA (this work), HNO_3_,^47^ HCl,^49^ and H_2_SO_4_,^46^ by undiluted TBP at room temperature with an initial O/A ratio of 1.

The extraction of mineral acids by TBP has been widely studied.^16,46–49^ For comparison with MSA, focus is placed on other strong acids (HNO_3_, HCl, and H_2_SO_4_), which are the most common acids for hydrometallurgical applications. H_2_SO_4_ is structurally similar to MSA, differing only by one hydrogen atom replaced by a methyl group. Hanson and Patel showed that only the first ionisation of H_2_SO_4_ occurs in the organic phase, suggesting a similar extracted species.^46^ Both acids exhibit negligible extraction below 2 mol L^−1^, but the distribution isotherm of MSA rises more sharply than that of H_2_SO_4_ between 2 and 8 mol L^−1^ acid, indicating better extraction by undiluted TBP (Fig. 9). Both systems show third-phase formation at high acid concentrations with diluted TBP and show exothermic behaviour with varying temperature.

HNO_3_ is generally extracted more efficiently, with its distribution isotherm above that of MSA. HNO_3_ extraction flattens at higher HNO_3_ concentrations in the feed, whereas MSA extraction still increases under similar conditions. This difference likely reflects the greater water uptake by TBP loaded with MSA, and the structural differences between molecular HNO_3_ and MSA that are extracted by the (TBPH^+^)(X^−^) adduct at these conditions. Like MSA, HNO_3_ extraction decreases with TBP dilution and shows third-phase formation at high acid concentrations. Temperature effects are similar, with declining extraction at higher temperatures. Zhang *et al.* reported that salts such as Ca(NO_3_)_2_ enhance HNO_3_ extraction, similar to the salting-out effect observed for MSA with Ni(CH_3_SO_3_)_2_.^48^ Precipitation at high acid and salt concentrations was also reported for both systems.

HCl extraction surpasses that of MSA at low acid concentrations but remains lower once the raffinate exceeds 5 mol L^−1^ acid in the raffinate. Kertes observed significant coextraction of water with HCl, far exceeding that in H_2_SO_4_ and HNO_3_ systems.^49^ This coincides with a significant increase in (O/A)_e_ for the HCl system, but no phase miscibility was observed. Coextraction of water with MSA is comparable to that in HCl systems, but (O/A)_e_ increases more rapidly with increasing MSA extraction, leading to phase miscibility starting from 11.8 mol L^−1^ MSA in the feed.

Overall, the following order of extraction efficiencies of strong acids by TBP could be identified: HNO_3_ > HCl ≈ MSA > H_2_SO_4_.

### 3.2 Thermodynamic model development

The experimental data collected in this study were subsequently used to expand the OLI-MSE thermodynamic model for acid extraction by TBP developed in previous publications.^16,33^ In these papers, the TBP–H_2_O chemistry present in the general OLI database was expanded by the introduction of a new species, namely protonated TBP (TBPH^+^). The phase behaviour was fitted through binary interaction parameters between the solvents (H_2_O and *n*-dodecane) and extractant species (TBP and TBPH^+^). Then, acid extraction was obtained in the model based on eqn (1) and (2), by fitting interaction parameters between the extractant and acid species. The latter includes H_3_O^+^, acid anions (CH_3_SO_3_^−^), and molecular acid (CH_3_SO_3_H). A more complete description of the thermodynamic model can be found in the accompanying publications. Large deviations between calculations and experimental data were observed when applying the initial model to the MSA–TBP extraction system. As expected, MSA-specific interaction parameters must be included to expand the thermodynamic model to these systems.^16^

A systematic approach was followed for expanding the acid–TBP model to include MSA extraction. Modelling started with the simplest system (MSA extraction by undiluted TBP at 25 °C) and the minimum number of interaction parameters required to obtain a good fit. Next, a more complex system was introduced, and new binary interaction parameters were fitted without changing the previously fitted parameters. This process was repeated until all the data were fitted. Conditions where a third phase was observed were excluded from the optimisation datasets, as the OLI-MSE framework is limited to two liquid phases. As a result, the final thermodynamic model cannot be used to predict third-phase formation. Additionally, care was taken to match the parameter type closely with the underlying chemistry.^31^ Species were used to represent bonds with significant covalent character (*e.g.* formation of TBPH^+^), short-range interactions were used for the solvation of neutral species, mid-range interactions were introduced when specific interactions between a neutral/charged or charged/charged species are encountered, and long-range interactions are present without introducing interaction parameters to account for the electrostatic effects.

First, mid-range binary energy (*b*_*ij*,0_ and *c*_*ij*,0_, eqn (7)) and density (*d*_*ij*,1_, eqn (8)) interaction parameters between CH_3_SO_3_^−^ and the extractant species were fitted to data from the MSA–undiluted TBP system below 100 %*L* (SI, Table S1). These data include Δ*G*_t_(MSA), *ρ*_o_, log([H_2_O]_o_), and (O/A)_e_ measurements. The Δ*G*_t_(MSA) data was given the highest weight as acid extraction is the most important property of this system. After a reasonable fit was obtained through the extraction of MSA as the (TBPH^+^)(CH_3_SO_3_^−^) adduct, data exceeding 100 %*L* were introduced (SI, Table S1). With these data, the extraction of molecular MSA could be fitted using short-range binary energy interaction parameters (*a*_*ij*,0_ and *a*_ji,0_, eqn (9)) between CH_3_SO_3_H and TBPH^+^. These interactions represent the solvation of CH_3_SO_3_H by TBPH^+^.7
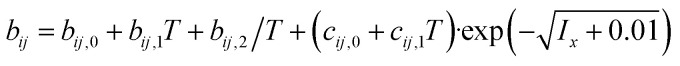
8
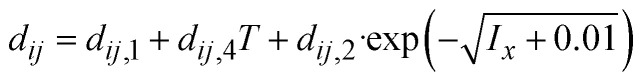

9 *a*_*ij*_ = *a*_*ij*,0_ + *a*_*ij*,1_*T*

These equations reflect the interactions between species *i* and *j*. *T* is the temperature in Kelvin, and *I*_x_ is the ionic strength on a mole fraction scale.

The mid-range parameters *b*_*ij*,0_ and *c*_*ij*,0_ between CH_3_SO_3_^−^ and TBPH^+^ capture the weak, non-ideal interactions that stabilise the protonated (TBPH^+^)(CH_3_SO_3_^−^) ion pair. These interactions account for part of the observed differences in extraction efficiency among acid anions by TBPH^+^. For loadings below <100 %*L* , additional contributions arise from the acid–base equilibria and the activity of H_3_O^+^ and the acid anions in the aqueous phases. These interactions are represented by the interaction parameters in the general OLI-MSE database, which are typically fitted to water activity, solubility and speciation data. The *b*_*ij*,0_ and *c*_*ij*,0_ interaction parameters between CH_3_SO_3_^−^ and TBP describe secondary (outer-sphere) stabilisation of the (TBPH^+^)(CH_3_SO_3_^−^) adduct through association with free TBP.

The observed changes in organic phase density could not be explained solely by differences in pure liquid densities between the TBP–*n*-dodecane mixture and the extracted H_2_O and MSA. Specific interactions between CH_3_SO_3_^−^ and TBPH^+^ also influence the occupied volume, as evidenced by the need to introduce binary density interaction parameters (Table 2).

**Table 2:** Optimised short- and mid-range binary interaction parameters in the OLI-MSE framework for the MSA–TBP thermodynamic model

Species	Short-range^a^		Mid-range		
	*a*_*ij*_	*a*_*ji*_	*b*_*ij*_ b	*c*_*ij*_ b	*d*_*ij*_ c
CH_3_SO_3_H–TBP (2)			−1572.99		
CH_3_SO_3_H–TBPH^+^ (0)	−12301.6	−22105			
CH_3_SO_3_H–TBPH^+^ (1)	49.3021	52.2635			
CH_3_SO_3_^−^–TBP (0)			−1.69792	−7.91831	
CH_3_SO_3_^−^–TBPH^+^ (0)			2.99305	−31.2443	
CH_3_SO_3_^−^–TBPH^+^ (1)			1.48588 × 10^−2^		5.34491 × 10^−2^
CH_3_SO_3_^−^–TBPH^+^ (2)					−0.714789
CH_3_SO_3_^−^–TBPH^+^ (4)					1.17248 × 10^−3^
CH_3_SO_3_^−^–dodecane (0)			−32.90930	16.99850	

a Short-range binary energy interaction parameters: see eqn (9).

b Mid-range binary interaction parameters: see eqn (7).

c Mid-range binary density parameter: see eqn (8).

Finally, a repulsive temperature-dependent binary energy interaction parameter (*b*_*ij*,2_, eqn (7)) between CH_3_SO_3_H and TBP was introduced to avoid the extraction of molecular MSA at low %*L*. This is common practice for models using the OLI-MSE framework. Since acid–base reactions are extremely efficient, it is experimentally impossible to determine the negligible amount of CH_3_SO_3_H in organic phases where free TBP is still present. As no non-zero experimental values exist for extracted CH_3_SO_3_H under these conditions, the *b*_*ij*,2_ interaction parameter cannot be uniquely determined from data alone. Nevertheless, assigning a sufficiently repulsive value ensures that the model does not predict molecular-acid extraction before TBP is protonated, which is consistent with the experimentally observed behaviour.

Next, the effects of TBP dilution were modelled by fitting *b*_*ij*,0_ and *c*_*ij*,0_ between CH_3_SO_3_^−^ and *n*-dodecane to data on the diluted TBP systems (SI, Table S2). These interaction parameters capture the more-than-stoichiometric decrease in MSA extraction upon dilution with *n*-dodecane (Fig. 4). Finally, temperature effects were included in the model by fitting temperature-dependent binary interaction parameters to data of systems at temperatures other than 25 °C (SI, Table S3). In the OLI-MSE framework, this is performed by expanding the expressions for the short-range energy (*a*_*ij*_), mid-range energy (*b*_*ij*_), and mid-range density parameters (*d*_*ij*_), following eqn (7)–(9). The introduction of temperature-dependent interaction parameters reflects the experimentally observed non-ideal temperature effects, evidenced by the variation of enthalpy and entropy of transfer (Δ*H*_t_ and Δ*S*_t_) with changing feed composition (Table 1). An overview of all optimised binary interaction parameters can be found in Table 2.

**Fig. 10:**
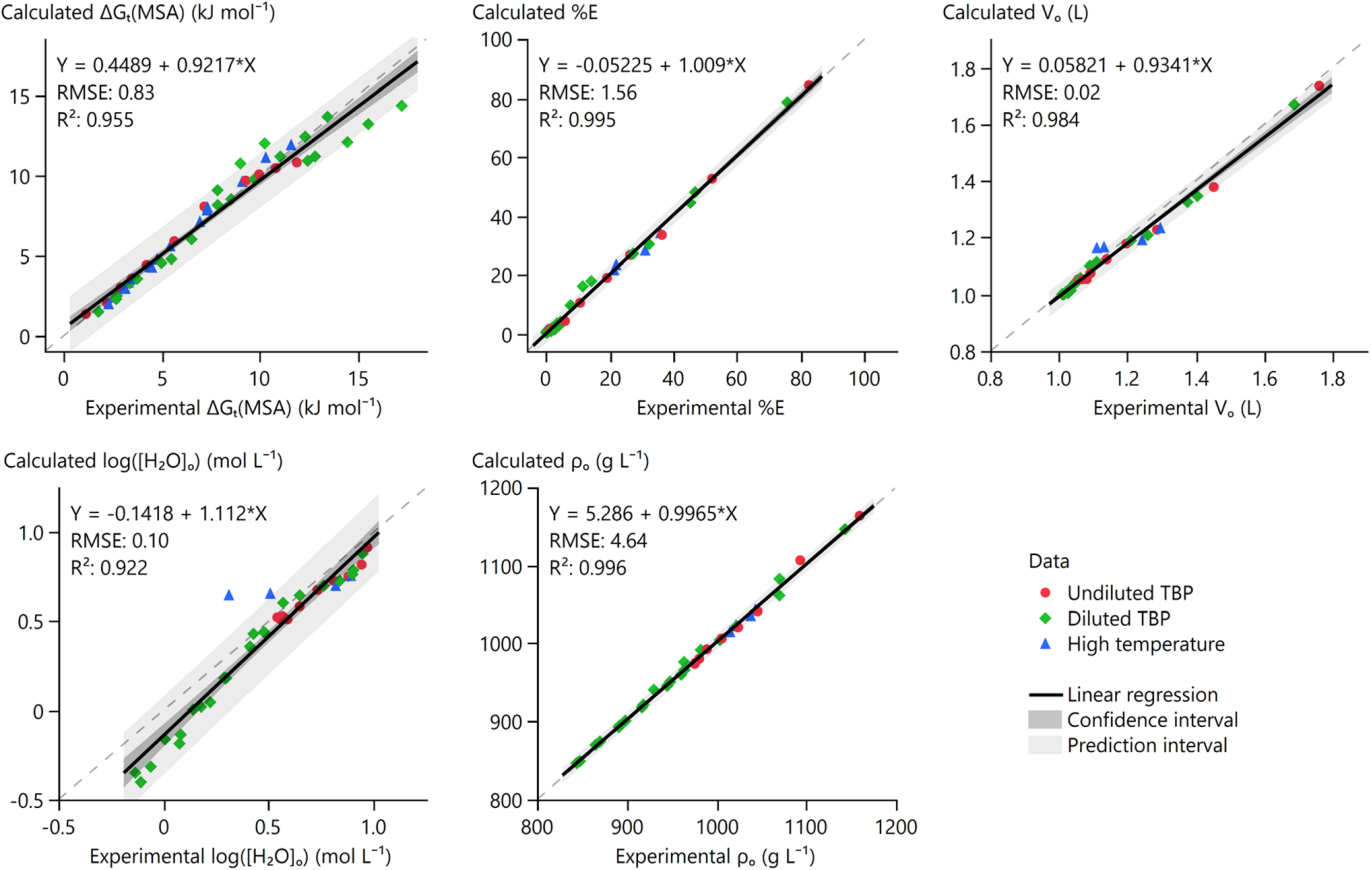
Quality of fit (QoF) graphs for the properties used to build the MSA–TBP thermodynamic model.

The performance of the model for key properties was quantified through Quality of Fit (QoF) graphs across different conditions, including variations in MSA concentration, extractant concentration, and temperature (Fig. 10). These plots show calculated against experimental values, with the bisector (*y* = *x*, grey dotted line) indicating perfect agreement. A linear regression of the data is plotted together with its 95% confidence and prediction interval. A lack of fit is found when (part of) the bisector lies outside the confidence interval. The prediction interval shows the range where a future calculated value is likely to lie for a given experimental point, reflecting uncertainty in individual predictions.

The experimental data are generally fitted well. No lack of fit is found for Δ*G*_t_(MSA), except at the highest values. These high values correspond to very low extractions of MSA by diluted TBP that are more difficult to accurately quantify experimentally. This translates to an accurate fit of the %*E* values, with a narrow prediction interval of ±3.1% around the mean at the 95% confidence level (RMSE × 1.96). This error falls within typical experimental uncertainty margins for SX measurements. Hence, calculating MSA extraction by TBP proves possible between 0.1–9.9 mol L^−1^ MSA in the feed, 50–100 wt% TBP in the solvent, and 24–77 °C.

Some lack of fit is observed for log([H_2_O]_o_), specifically at lower water contents. This deviation is likely related to both model limitations and a typically higher uncertainty associated with Karl Fischer titrations in complex solutions. Two data points of the high-temperature series (59 and 77 °C) deviate from the trend, as the model significantly overestimates [H_2_O]_o_ under these conditions. The initial acid–TBP model was not optimised for [H_2_O]_o_ data at elevated temperatures, and no temperature-dependent corrections were applied to the H_2_O–TBPH^+^ interaction parameters.^16^ Nevertheless, the model provides a reasonable estimate of [H_2_O]_o_, well within an order-of-magnitude range of the experimental values.

Extraction of water and MSA causes a significant increase in the *V*_o_ and the connected (O/A)_e_ in both the model and the experiments. Overall, a good fit of *V*_o_ is obtained with a small underestimation of *V*_o_ in the model compared to the experimental observations. This leads to the still reasonably calculated value of 1.69 ± 0.04 (95% certainty) for the highest experimental values of 1.75.

Finally, MSA is significantly denser than TBP, resulting in an increase in *ρ*_o_ upon MSA extraction. The QoF graph for *ρ*_o_ shows no lack of fit and has a 95% prediction interval of ±9.09 g L^−1^ around the mean. Thus, the density of the organic phase can be calculated accurately over the whole range. Graphs comparing model fits with individual experimental data can be found in the SI (Fig. S1–S3).

### 3.3 Model validation and process simulation

#### 3.3.1 Validation with system containing Ni(CH_3_SO_3_)_2_

The predictive performance of the thermodynamic model was evaluated using the experimental Ni(CH_3_SO_3_)_2_ datasets (SI, Table S4) that were not included in the parameter optimisation. This approach ensures that the accuracy of the model is tested under conditions beyond the range of fitting data. Validation is focused on the equilibrium extraction of MSA, as this represents the most important property for process design and the key outcome the model is intended to calculate.

**Fig. 11:**
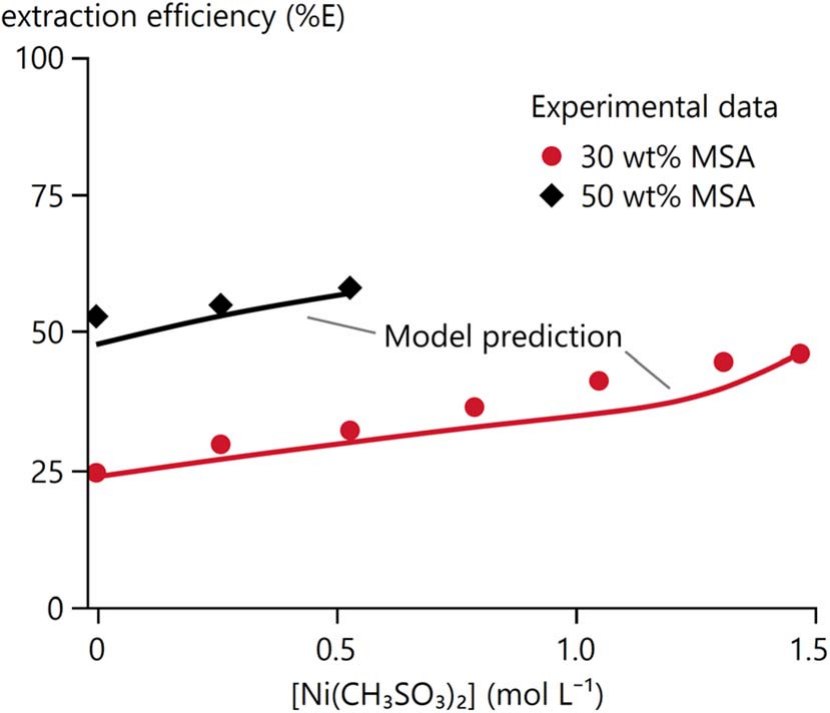
Model prediction of experimental data on the MSA–Ni(CH_3_SO_3_)_2_–H_2_O–TBP system at 25 °C from Table S4.

The Ni(CH_3_SO_3_)_2_ chemistry was not included in the general OLI-MSE database, but a private OLI-MSE database has been built by Derison *et al.*^43^ This database was combined with the general OLI, acid–TBP, and MSA–TBP databases to calculate the equilibrium in this system.^16^Fig. 11 shows good qualitative agreement between predicted and experimental MSA extraction, while similar plots for other equilibrium properties are provided in the SI (Fig. S4). These properties are generally well predicted, with only minor deviations. A slightly steeper increase in predicted *ρ*_o_ is observed with increasing concentration of Ni(CH_3_SO_3_)_2_ compared to experimental values, and a modest underestimation of *V*_o_ and log([H_2_O]_o_) across the entire concentration range can be detected.

Some extraction of nickel(ii) was initially predicted *via* Ni_2_(OH_3_)^+^ distribution. Since this was not observed experimentally, a repulsive binary energy interaction parameter between Ni_2_(OH)_3_^+^ and *n*-dodecane (*b*_*ij*,2_, = −7626.785, eqn (7)) was introduced to suppress nickel(ii) extraction in the calculations. Such a repulsive interaction parameter between an ion and an apolar molecule is commonly set in the general MSE-OLI database.

#### 3.3.2 Process simulation for MSA recovery

Extraction of MSA by TBP is relatively weak, meaning that a single-stage extraction at an initial O/A of 1 yields only low acid recovery. Efficiency can be significantly enhanced by optimising the O/A ratio and implementing a multi-stage, continuous, counter-current setup in mixer-settlers.^13^ After extraction, a stripping step is required to recover MSA from the loaded organic phase. Developing and optimising such a process experimentally would be time-consuming, so calculations with the thermodynamic model could be helpful. Because the underlying chemistry remains constant and the same equilibria apply, the model is not restricted to a single O/A ratio and can be applied to both extraction and stripping steps. When integrated into process simulation software, the model enables the calculation of complete mixer-settler batteries for both extraction and stripping.

A full process flowsheet was developed and optimised in OLI Flowsheet for MSA recovery from aqueous solutions containing Ni(CH_3_SO_3_)_2_, leveraging the validated thermodynamic model for this system. A diagram of this flowsheet is provided in the SI (Fig. S5). The feed solution contained 1 mol L^−1^ free MSA and 1.5 mol L^−1^ Ni(CH_3_SO_3_)_2_, which could represent a nickel(ii)-containing leachate with excess acid. Some remaining acid could still be present in real leachates, as excess acid improves leaching efficiency and rate.^1^ Extraction was simulated at 20 °C. A lower temperature would improve extraction further (Fig. 7), but would be less practical in operation. Stripping was simulated with hot water at 75 °C to enhance efficiency. A total of 8 extraction and 2 stripping stages were sufficient to achieve a high overall recovery of MSA.

**Fig. 12:**
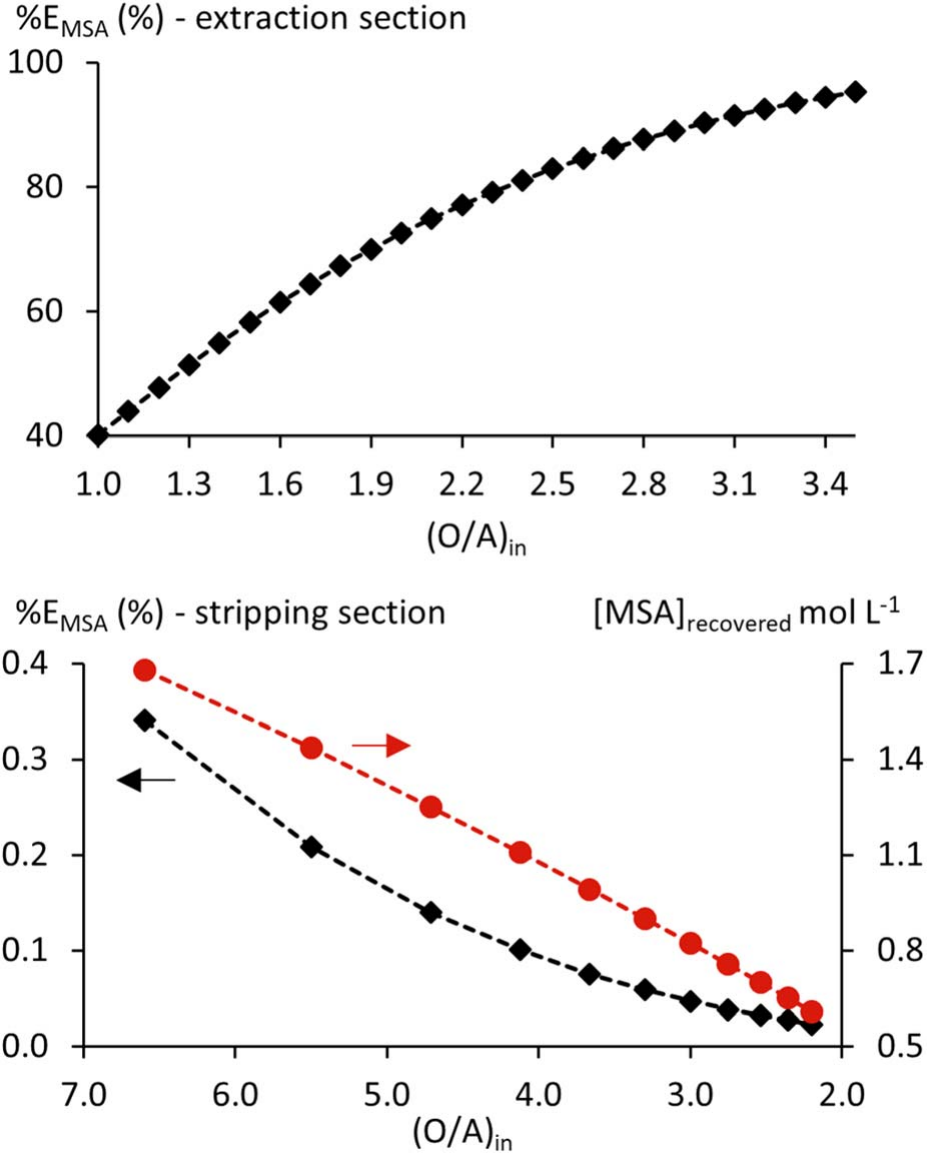
Extraction efficiency (%*E*, ◆) for extraction (top) and stripping (bottom) sections of the MSA recovery flowsheet (Fig. S5) with varying initial O/A ratios. Recovered [MSA] is given for the stripping section on the right *y*-axis (●).

Only 40% MSA extraction was calculated at an initial O/A of 1 for the extraction section, whereas complete stripping was achieved in three stages at the same phase ratio. This represents a significant improvement compared to the mere 3% extraction predicted for a single stage without dissolved salts (Fig. 1), highlighting the efficacy of the combined salting-out effect and multi-stage operation. A sensitivity analysis was performed to optimise the extraction efficiency by varying the flow rate of the solvent between 1 and 3.5 L h^−1^ at an initial feed flow rate of 1 L h^−1^. Higher solvent flow rates would not be practical from a process control standpoint.^13^ Extraction can be increased significantly by increasing O/A, but the increase flattens at the highest phase ratios (Fig. 12). A phase ratio of 3.3 was selected as a practical compromise between efficiency increase and MSA dilution, and the practical operation of mixer-settlers. At this phase ratio, the simulation resulted in 94% MSA extraction after 8 stages.

A second sensitivity analysis varied the stripping water flow between 0.5 and 1.5 L h^−1^, corresponding to a stripping O/A from 6.6 to 2.2. More than 99.9% stripping was calculated at an initial stripping O/A of 4.13, yielding a recovered [MSA] of 1.11 mol L^−1^ (Fig. 12). Overall, the calculated recovery reached 94%, with the recovered [MSA] exceeding that of the feed. Even at a reduced initial stripping O/A of 3 for practical reasons, the recovered concentration remained high at 0.82 mol L^−1^. For both initial stripping O/A, the recovered MSA could be recycled to the leaching stage with minimal upconcentration, depending on process requirements.

Beyond extraction and stripping efficiencies, the simulation also calculates other equilibrium properties (SI, Fig. S5). For example, the calculated *V*_o_ increase is limited (+1%) across the extraction section due to the relatively low [MSA]_in_ and a large initial O/A. Simultaneously, the aqueous phase volume decreased by 6%, raising the nickel(ii) concentration in the raffinate to 1.59 mol L^−1^ without any nickel(ii) extraction or precipitation. Losses of TBP to the raffinate and recovered MSA stream were minimal, with calculated concentrations of 7–9 × 10^−4^ mol L^−1^ in both cases.

## 4 Conclusions

This work describes a predictive, unified thermodynamic model for the recovery of methanesulphonic acid (MSA) by tri-*n*-butyl phosphate (TBP), based on experimental SX data and implemented within the OLI Mixed-Solvent Electrolyte framework. The model captures the dominant extraction mechanism through TBPH^+^·CH_3_SO_3_^−^ ion pair formation, and the additional uptake of molecular MSA at high extractant loadings. Across a wide range of acid concentrations (0.1–9.9 mol L^−1^), TBP concentrations (50–100 wt%), and relevant operation temperatures (24–77 °C), the optimised interaction parameters reproduce MSA Gibbs free energy of transfer, MSA extraction efficiency, organic-phase density, and volume changes within experimental uncertainty, while also providing a good estimate of the water uptake by the organic phase. The model further reflects the exothermic nature of MSA extraction and its decrease with increasing temperature. Dilution in *n*-dodecane reduces extraction more than stoichiometrically and can induce third-phase splitting at high acid and low TBP concentrations. Ni(CH_3_SO_3_)_2_ causes a modest salting-out enhancement of MSA extraction without co-extraction of nickel(ii), which the thermodynamic model accurately predicts. Comparing MSA extraction data with literature data on the extraction of other strong acids by TBP reveals extraction efficiencies following HNO_3_ > HCl ≈ MSA > H_2_SO_4_. Process simulations using counter-current mixer-settlers demonstrate practical relevance, as an overall MSA recovery of 94% is calculated with optimised phase ratios for 8 extraction stages and 2 hot water stripping stages. These findings position MSA as a viable and more sustainable alternative to sulfuric acid in hydrometallurgical SX, combining more efficient recovery by TBP with a greener profile.

## Author contributions

Rayco Lommelen: conceptualisation, formal analysis, data curation, methodology, validation, visualisation, supervision, writing – original draft. Charlotte Lempereur: data curation, formal analysis, investigation, methodology, validation, visualisation, writing – review & editing. Koen Binnemans: conceptualisation, funding acquisition, resources, supervision, writing – review & editing.

## Conflicts of interest

The authors declare that they have no known competing financial interests or personal relationships that could have appeared to influence the work reported in this paper.

## Supplementary Material

RA-016-D6RA00938G-s001

## Data Availability

The data supporting this article have been included as part of the supplementary information (SI). *n*-dodecane. Supplementary information: detailed thermodynamic model fits to the experimental data, a screenshot of the solvent extraction simulation flowsheet, and a unit conversion table for TBP and *n*-dodecane. See DOI: https://doi.org/10.1039/d6ra00938g.
