# Supplementary material for: Thermodynamic model for methanesulphonic acid recovery by tri-n-butyl phosphate
Source: RSC Adv. 2026 Apr 7;16(20):18154–65. doi: 10.1039/d6ra00938g (PMC13055884; doi:10.1039/d6ra00938g)
Supplement: RA-016-D6RA00938G-s001 [file RA-016-D6RA00938G-s001.pdf]

Supplementary Information (SI) for RSC Advances.
This journal is © The Royal Society of Chemistry 2026

Thermodynamic model for methanesulfonic acid recovery by tri-n-butyl phosphate

Rayco Lommelen,[a*] Charlotte Lempereur,[a†] and Koen Binnemans[a]

[a]KU Leuven, Department of Chemistry, Celestijnenlaan 200F, P.O. box 2404, B‑3001 Leuven, Belgium

* Corresponding author: Email: rayco.lommelen@kuleuven.be

Supplementary Information (SI)

† New address: Université Grenoble Alpes, Université Savoie Mont-Blanc, CNRS, Grenoble INP, LEPMI, 38000 Grenoble, France

1. Experimental details and results

Explanation for abbreviations in tables:

- a = aqueous phase
- o = organic phase
- in = initial
- e = equilibrium
- ρ = density
- *O/A* = organic to equation volumetric ratio
- D_{MSA} = distribution ratio = $\frac{[\mathrm{MSA}]_{\mathrm{o,e}}}{[\mathrm{MSA}]_{\mathrm{a,e}}}$
- E_{MSA} = extraction efficiency = $\frac{[\mathrm{MSA}]_{\mathrm{o,e}} \cdot (O/A)_e}{[\mathrm{MSA}]_{\mathrm{aq,in}}} \cdot 100$
- L_{MSA} = extractant loading = $\frac{[\mathrm{MSA}]_{\mathrm{o,e}} \cdot (O/A)_e}{[\mathrm{TBP}]_{\mathrm{o,in}}} \cdot 100$

The following equation can be used to convert the TBP concentration in *n*-dodecane from wt% to vol%:

$$[\mathrm{TBP}]\ (vol\%) = \frac{[\mathrm{TBP}]\ (wt\%)\ /\ \rho_{TBP}}{[\mathrm{TBP}]\ (wt\%)\ /\ \rho_{TBP} + [\mathrm{dodecane}\ ]\ (wt\%)\ /\ \rho_{dodecane}}$$

with ρ_{TBP} = 973 g L^{-1} and $\rho_{dodecane}$ = 750 g L^{-1}.

Table S1. Experimental details for MSA–100 vol% TBP system collected at 25 °C with O/A_{in} = 1.

$[MSA]_{a,in}$ mol L^{-1}	$[MSA]_{a,e}$ mol L^{-1}	$[MSA]_{o,e}$ mol L^{-1}	ρ_o g mL^{-1}	$[H_2O]_o$ mol L^{-1}	$(O/A)_e$	D_{MSA}	E_{MSA} %	L_{MSA} %
0.00	0.00	0.00	0.976	3.70	1.13			
0.10	0.10		0.976	3.60				
0.25	0.25	0.00	0.976	3.48	1.18	0.008	0.93	0.1
0.49	0.51	0.01	0.976	3.55	1.12	0.013	1.39	0.2
0.74	0.76	0.01	0.976	3.69	1.13	0.018	1.97	0.4
0.98	1.01	0.02	0.977	3.63	1.13	0.024	2.57	0.7
1.96	1.96	0.11	0.981	3.89	1.15	0.056	5.95	3.2
2.96	2.79	0.29	0.989	4.44	1.20	0.104	10.69	8.7
3.94	3.60	0.66	1.006	5.40	1.33	0.184	19.14	20.9
4.93	4.35	1.09	1.024	6.42	1.49	0.249	26.37	35.9
5.92	5.34	1.67	1.046	7.57	1.80	0.313	36.39	59.5
7.89	6.82	2.83	1.093	8.76	2.65	0.415	52.12	117
9.86	7.20	4.62	1.160	9.32	7.35	0.642	82.53	225.

Table S2. Experimental details for MSA–TBP–dodecane system collected at 25 °C with O/A_{in} = 1. $[n\text{-dodecane}]_{o,in}$ (wt%) = 100% - $[TBP]_{o,in}$

$[MSA]_{a,in}$ mol L^{-1}	$[TBP]_{o,in}$ wt%	$[MSA]_{a,e}$ mol L^{-1}	$[MSA]_{o,e}$ mol L^{-1}	ρ_o g mL^{-1}	$[H_2O]_o$ mol L^{-1}	$(O/A)_e$	D_{MSA}	E_{MSA} %	L_{MSA} %
0.25	50.2			0.844	0.87				
0.25	60.2	0.25	0.00	0.865	1.21	1.04	0.001	0.1	0.0
0.25	69.6	0.25	0.00	0.891	1.51	1.05	0.002	0.2	0.0
0.25	79.5	0.25	0.00	0.917	1.99	1.06	0.003	0.3	0.0
0.25	89.8	0.25	0.00	0.945	2.62	1.09	0.006	0.6	0.0
0.25	95.0	0.25	0.00	0.961	3.05	1.10	0.007	0.7	0.1
1.97	50.2	1.98	0.01	0.844	0.73	1.02	0.004	0.4	0.6
1.97	60.2	2.06	0.01	0.865	1.02	1.04	0.007	0.7	0.7
1.97	69.6	2.07	0.02	0.892	1.38	1.04	0.012	1.2	1.1
1.97	79.5	1.97	0.04	0.919	1.94	1.07	0.020	2.0	1.5
1.97	89.8	2.05	0.07	0.948	2.58	1.09	0.032	3.5	2.2
1.97	95.0	2.02	0.09	0.964	3.00	1.11	0.042	4.5	2.7
3.91	50.2	3.93	0.06	0.848	0.78	1.03	0.016	1.6	4.0
3.91	60.2	3.94	0.10	0.870	1.19	1.06	0.026	2.7	5.5
3.91	69.6	3.67	0.16	0.899	1.67	1.07	0.042	4.1	7.0
3.91	79.5	3.90	0.28	0.930	2.68	1.13	0.073	7.7	11.1
3.91	89.8	3.76	0.41	0.964	3.71	1.12	0.110	11.5	14.3
3.91	95.0	3.70	0.50	0.983	4.45	1.25	0.135	14.2	16.4
5.87	89.8	5.94	1.33	1.004	5.78	1.53	0.223	27.4	50.9
5.87	95.0	5.82	1.51	1.022	6.89	1.70	0.259	32.4	56.1
7.82	89.8	7.64	2.58	1.070	7.93	2.20	0.337	45.3	112
7.82	95.0	7.61	2.61	1.070	7.99	2.36	0.342	46.8	108
9.78	95.0	8.84	4.38	1.143	8.86	5.42	0.496	75.7	219

Table S3. Experimental details for MSA–100 vol% TBP system with O/A_{in} = 1.

$[MSA]_{a,in}$ mol L^{-1}	T °C	$[MSA]_{aq,e}$ mol L^{-1}	$[MSA]_{org,e}$ mol L^{-1}	$[H_2O]_{org}$ mol L^{-1}	$(O/A)_e$	D_{MSA}	E_{MSA} %	L_{MSA} %
2.02	24	2.04	0.11		*1.13* [a]	0.052	*5.5* [a]	*3.1* [a]
2.02	40	2.13	0.06		*1.11* [a]	0.030	*3.4* [a]	*1.9* [a]
2.02	59	2.13	0.05		*1.11* [a]	0.024	*2.7* [a]	*1.5* [a]
2.02	77	2.14	0.04		*1.11* [a]	0.019	*2.1* [a]	*1.2* [a]
4.01	24	4.04	0.66		*1.38* [a]	0.163	*19* [a]	*21* [a]
4.01	40	4.12	0.52		*1.27* [a]	0.125	*14* [a]	*16* [a]
4.01	59	4.25	0.35		*1.21* [a]	0.081	*10* [a]	*10* [a]
4.01	77	4.27	0.35		*1.20* [a]	0.081	*10* [a]	*11* [a]
6.01	24	5.74	1.67	7.78	1.843	0.290	35.9	32.4
6.01	40	5.79	1.51	6.59	1.645	0.260	31.1	31.5
6.01	59	5.66	1.17	3.22	1.305	0.207	22.0	28.1
6.01	77	5.92	1.16	2.05	1.250	0.196	21.4	28.4
8.01	24	7.42	2.97		*2.56* [a]	0.400	*52* [a]	*114* [a]
8.01	40	7.49	2.83		*2.05* [a]	0.378	*45* [a]	*101* [a]
8.01	59	7.33	2.38		*1.71* [a]	0.324	*35* [a]	*78* [a]
8.01	77	7.15	2.32		*1.59* [a]	0.324	*33* [a]	*73* [a]

[a] Determined based on equilibrium volumes calculated by the thermodynamic model

Table S4. Experimental details for MSA–Ni(CH₃SO₃)₂–100 vol% TBP at 25 °C with O/A$_{in}$ = 1.

$[MSA]_{a,in}$ mol L^{-1}	$[Ni(MSA)_2]_{a,in}$ mol L^{-1}	$[MSA]_{aq,e}$ mol L^{-1}	$[MSA]_{org,e}$ mol L^{-1}	ρ_o g mL^{-1}	$[H_2O]_{org}$ mol L^{-1}	$(O/A)_e$	D_{MSA}	E_{MSA} %	L_{MSA} %
4.60	0.00	4.48	0.92	1.015	6.46	1.56	0.205	24.4	30.9
4.60	0.26	4.33	1.10	1.022	6.69	1.63	0.253	29.6	37.5
4.60	0.53	4.04	1.19	1.026	6.55	1.63	0.295	32.1	40.7
4.60	0.79	3.82	1.34	1.032	6.62	1.67	0.350	36.4	46.0
4.60	1.05	3.59	1.48	1.041	6.49	1.78	0.412	41.1	52.1
4.60	1.31	3.36	1.60	1.042	6.22	1.78	0.475	44.5	56.4
4.60	1.47	3.17	1.65	1.048	6.06	1.78	0.522	46.0	58.3
7.66	0.00	7.06	2.87	1.085	9.21	2.61	0.407	52.8	112
7.66	0.26	6.40	2.94	1.089	8.51	2.75	0.460	54.9	116
7.66	0.53	6.36	3.04	1.094	7.75	2.92	0.479	58.0	123

2. Thermodynamic model fits to experimental data

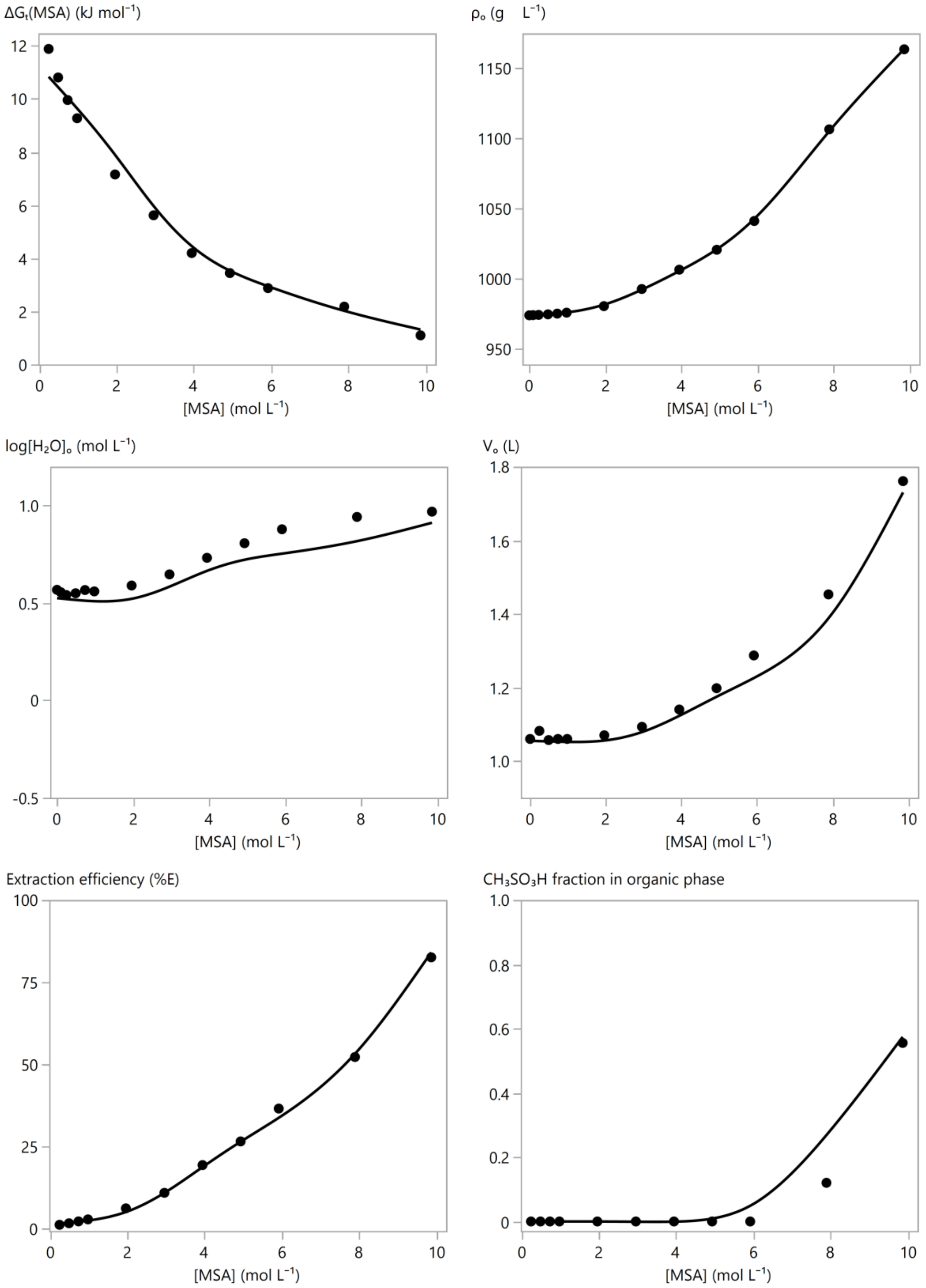

Figure S1. Model fit (lines) to experimental data (markers) with undiluted TBP at 25 °C from Table S1. Experimental CH_3SO_3H values are based on %L.

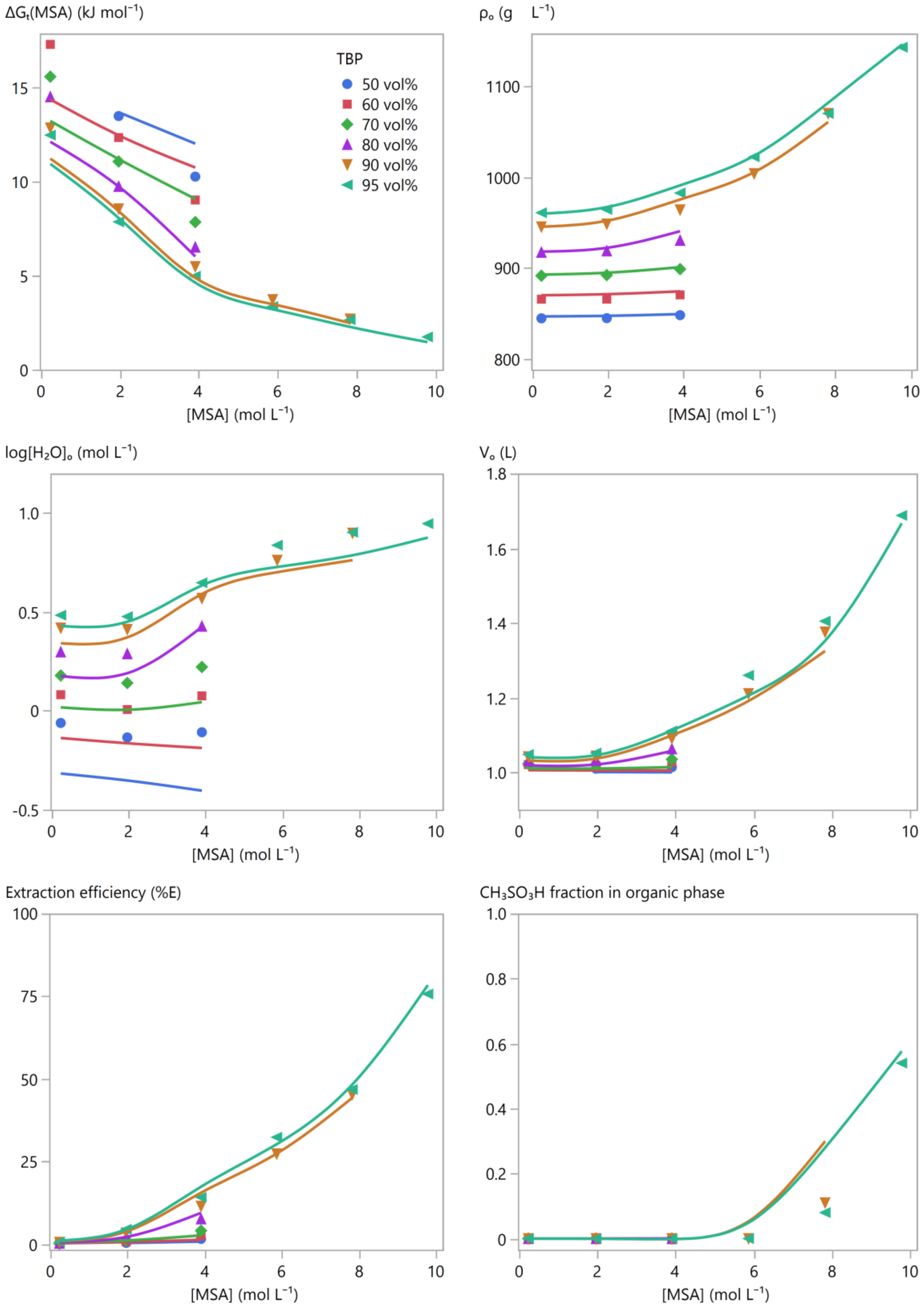

Figure S2. Model fit (lines) to experimental data (markers) with diluted TBP at 25 °C from Table S2. Experimental CH_3SO_3H values are based on %L.

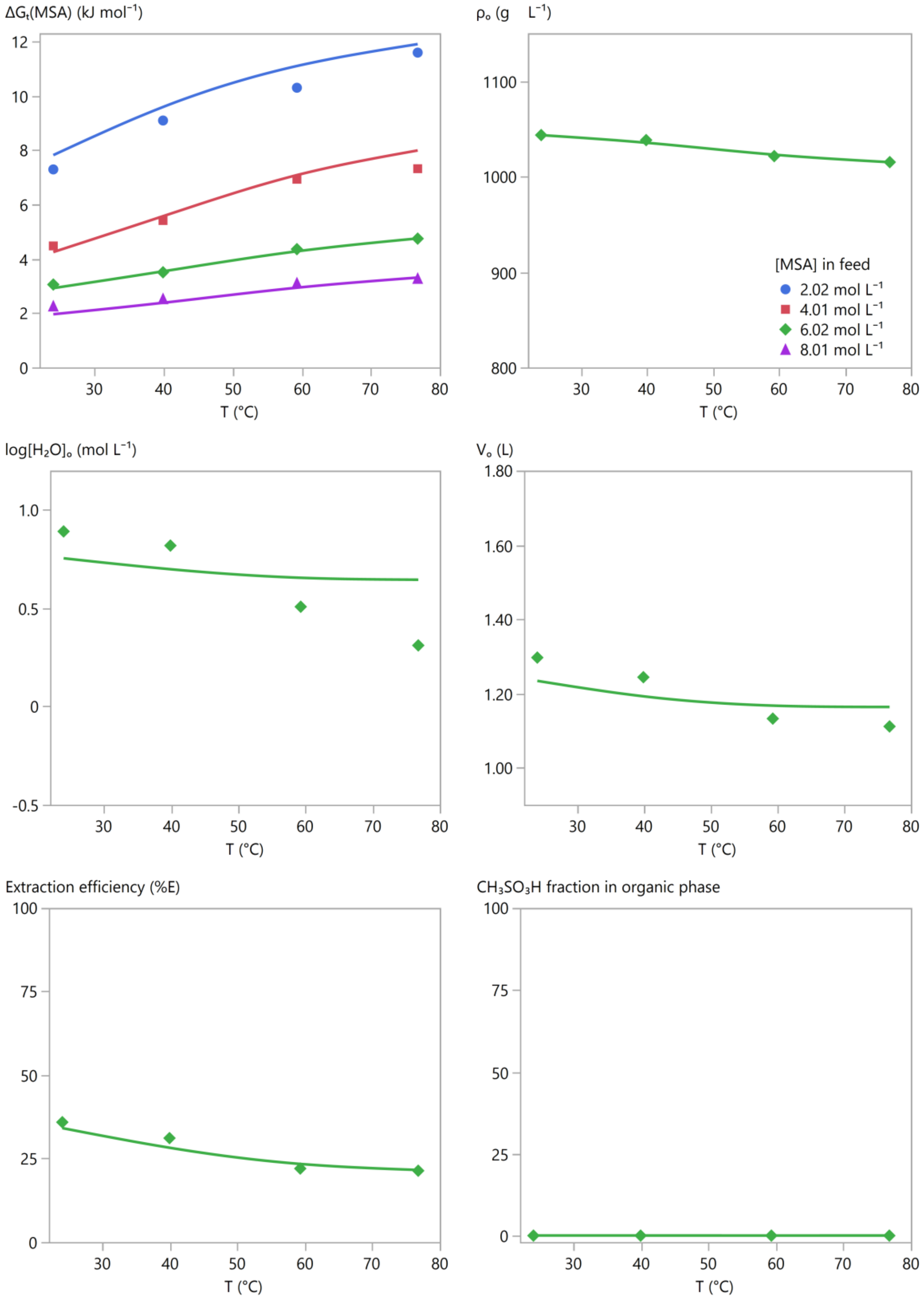

Figure S3. Model fit (lines) to experimental data (markers) with undiluted TBP at different temperatures from Table S1. Experimental CH_3SO_3H values are based on %L.

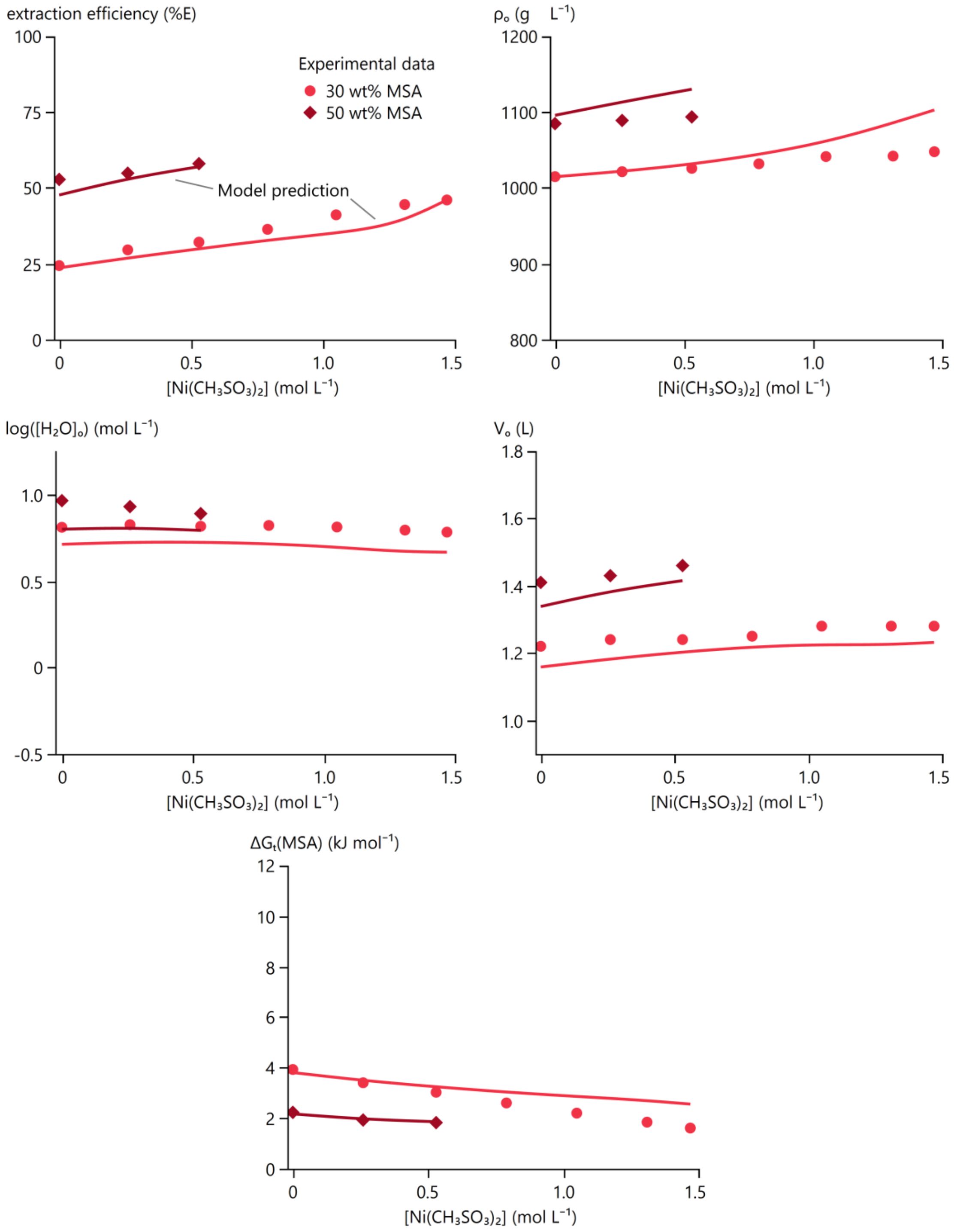

Figure S4. Model fit (lines) to experimental data (markers) on the MSA–Ni(CH_3SO_3)$_2$–H_2O–TBP system at 25 °C from Table S4.

3. Simulation of a solvent extraction process for MSA recovery

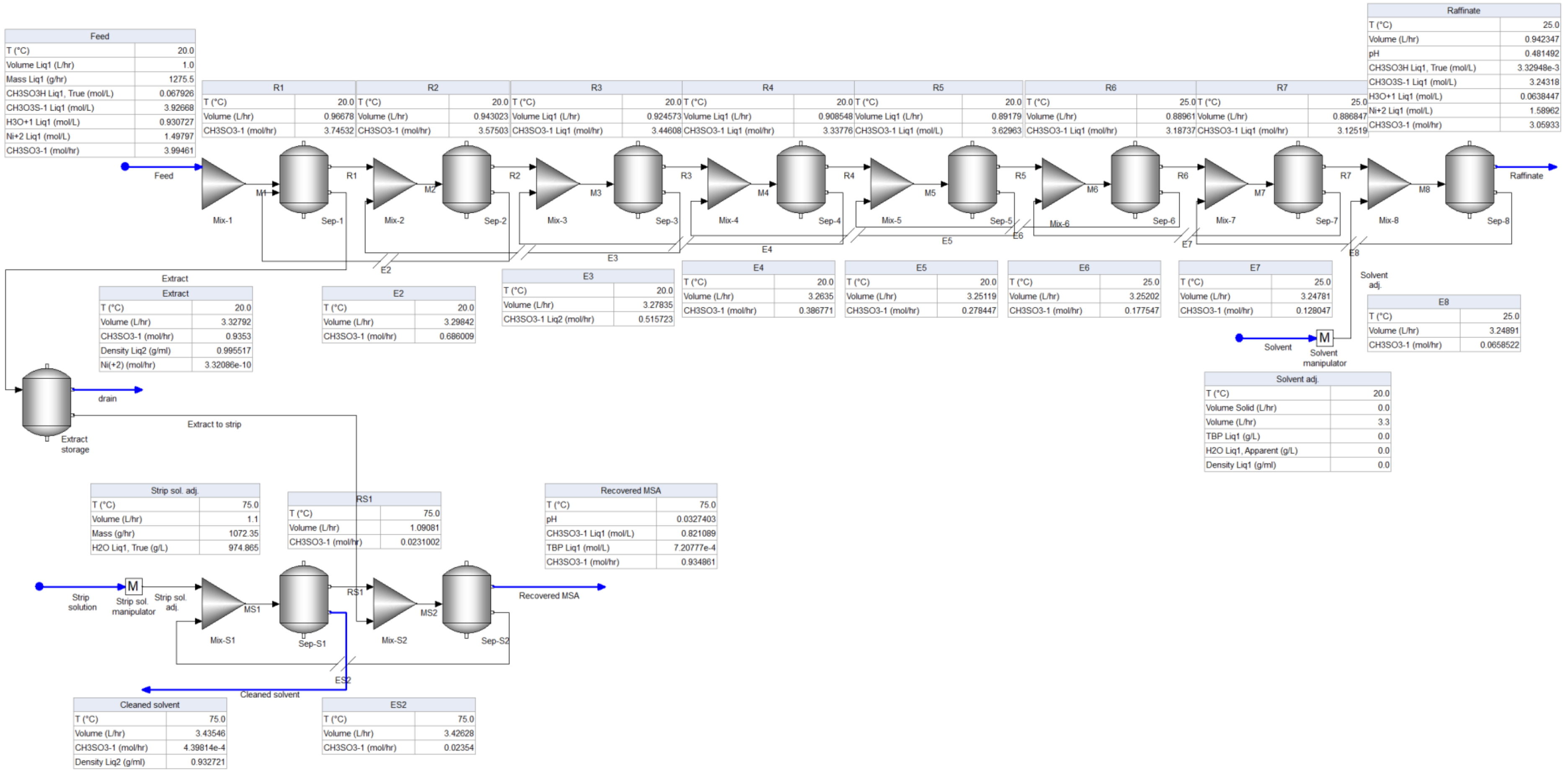

Figure S5. Flowsheet for MSA recovery with 8 extraction and 3 stripping stages developed in OLI Flowsheet version 12.0 from OLI Systems.

4. TBP and *n*-dodecane concentration conversion table

Table S5. Molar amounts and masses of tri-n-butyl phosphate (TBP), and n-dodecane (dod.) that must be mixed to obtain a certain concentration of TBP (in vol.%) in 1 litre of solvent.

**TBP (vol%)**	**n_{TBP} (mole)**	**in**	**$n_{dod.}$ (mole)**	**m_{TBP} (g)**	**in**	**$m_{dod.}$ (g)**
0	0		4.40	0		750
5	0.18		4.18	49		713
10	0.36		3.96	97		675
15	0.55		3.74	146		638
20	0.73		3.52	194		600
25	0.91		3.30	243		563
30	1.09		3.08	292		525
35	1.28		2.86	340		488
40	1.46		2.64	389		450
45	1.64		2.42	437		413
50	1.82		2.20	486		375
60	2.19		1.76	583		300
70	2.55		1.32	680		225
80	2.92		0.88	778		150
90	3.28		0.44	875		75
100	3.65		0.00	972		0
